# Pace and place tactics of top performers in the world's 3000 m steeplechase competitions

**DOI:** 10.1016/j.jesf.2026.200497

**Published:** 2026-07-28

**Authors:** Fangzhou He, Anning Guo, Mujuan Zhao, Jiqing Cui, Włodzimierz S. Erdmann

**Affiliations:** a Gdansk University of Physical Education and Sport, Department of Biomechanics and Sports Engineering, 1 Kazimierza Gorskiego street, Gdansk, 80-336, Poland; b Gdansk University of Physical Education and Sport, Faculty of Physical Culture, 1 Kazimierza Gorskiego street, Gdansk, 80-336, Poland; c Kyungpook National University, Department of Recreation and Sports, 2559, Gyeongsang-daero, Sangju, 37224, South Korea; d Weifang Institute of Technology, Physical Education and Sport, No. 9888, South Yunmen Street, Weifang, 262500, China

**Keywords:** 3000 m steeplechase, Race tactic, Velocity distribution, Place distribution, Elite athletes, Performance

## Abstract

**Aim:**

This study compared pacing and in-race place distribution patterns between successful and non-successful athletes in world-class men's and women's 3000 m steeplechase competitions, using official 100-m split data from heats and finals.

**Methods:**

A total of 339 performances from 144 athletes were collected from the Olympic Games and World Athletics Championships between 2021 and 2025. Indicators related to velocity and place were calculated, including the late-race deceleration index (LDI), coefficient of variation (CV), quotient of velocity deviations (QVD), CV_place_, and quotient of place deviations (QPD). Descriptive race-profile analyses were used to characterize velocity and place patterns. Linear mixed models and generalized linear mixed models were used for inferential analyses, with athlete identity included as a random intercept.

**Results:**

Both men and women displayed an overall fast–slow–fast pacing profile. Successful athletes showed higher peak velocity and lower LDI than non-successful athletes across all sex-by-round scenarios. In men, success was generally associated with greater global velocity modulation, particularly in the heats. In women, successful athletes showed better velocity maintenance and greater place variability, with earlier separation between successful and non-successful groups. In the GLMMs, lower LDI was the most consistent correlate of success, while the second success-related indicator differed by scenario: CV in men's heats, V_peak_ in women's finals, and CV_place_ in women's heats.

**Conclusions:**

Successful 3000 m steeplechase performances were characterized by superior overall velocity, greater peak velocity, and reduced late-race velocity loss. Late-race velocity maintenance appears to be a central feature of successful performance, whereas velocity modulation and place adjustment show specific patterns by sex and round.

## 1 Introduction

The 3000 m steeplechase is a track event that combines sustained endurance demands with the technical challenges of clearing hurdles and water jumps. Athletes must negotiate 28 barriers and 7 water jumps throughout the course of the race,^1^ and the event is often considered one of the most tactically complex disciplines among the middle-distance and long-distance events. Performance at the highest level depends not only on absolute running velocity, but also on how athletes distribute their effort and manage their place within the field throughout the race. A systematic comparison of pacing and placing tactics between successful and non-successful athletes in major championships may therefore provide valuable evidence to inform training prescriptions and race tactics. Previous work by Hanley and Williams^2^ analyzed the pacing profiles in the 3000 m steeplechase using data from the 2008 and 2016 Olympic Games. However, these analyses were limited by relatively small sample sizes, complex split definitions, and the restriction to only two Olympic editions, which constrains the generalizability and timeliness of their conclusions. Since 2021, some Olympic Games and World Championships organizers have begun to release official split times and intermediate race places at every 100 m, creating new opportunities for high-resolution, large-sample tactical analyses in this event.

From the perspective of theoretical and modeling studies on energy expenditure, even pacing is typically considered more economical and conducive to faster finishing times than frequent velocity changes,^3^ and this has been supported across a range of endurance disciplines.^4^ However, in actual championship racing, observational studies consistently show that medalists and finalists in middle-distance and long-distance events often adopt relatively conservative early or mid-race pacing and rely on late accelerations or end spurts, which are closely related to superior finishing rankings.^5^^,^^6^ The key reason is that, under championship conditions, athletes and coaches tend to prioritize higher finishing positions over faster finishing times.^7^ Compared with flat distance events, the presence of barriers and water jumps in the 3000 m steeplechase disrupts rhythm and amplifies velocity fluctuations, making pacing patterns more complex, particularly around barrier entries, exits, and water jumps. The way athletes adjust their velocity in these key sections has not yet been described in sufficient detail. In addition, previous analyses of Olympic and World Championship middle-distance races have shown that pacing profiles differ between qualifying rounds and finals, reflecting the different tactical demands of qualification and final placing.^5^ This suggests that pacing tactics may also differ between heats and finals in the 3000 m steeplechase.

As a head-to-head racing event, tactical decision-making in the 3000 m steeplechase is reflected not only in velocity changes but also in how athletes place themselves within the pack. Competitors continually adjust their rhythm and tactics in relation to the velocity and place of other runners, and these place changings are closely related to whether they ultimately succeed.^8^ Studies in middle-distance running have reported that maintaining a place within a group running at a moderate but not excessive pace in the early stages is beneficial for performance,^9^ whereas research in longer-distance events, such as the 5000 m and 10,000 m, has suggested that staying close to the lead pack from the beginning may be advantageous.^10^ These seemingly divergent findings imply that optimal place tactics differ across disciplines and race distances. In contrast, quantitative analyses of place tactics in the 3000 m steeplechase are scarce, with most previous work focusing on mean velocity or overall pacing curves. In principle, combining information on place distribution with velocity profiles should provide a more comprehensive picture of tactical decision-making in high-level steeplechase races, but this line of research has been constrained by the long-standing lack of publicly available intermediate place data.

Against this background, the present study uses high-resolution official split data from the Tokyo Olympic Games and the three most recent World Athletics Championships to describe and compare pacing and placing tactics in world-class men's and women's 3000 m steeplechase races, with particular attention to differences across sex and race round. This study aims to address the following questions: (1) In elite 3000 m steeplechase competition, how do successful and non-successful athletes differ in their patterns of velocity distribution and place distribution? (2) Are these differences consistent across heats and finals, and between men and women, or do they show scenario-specific patterns? Based on previous literature and the event's characteristics, we hypothesized that: (1) compared with non-successful athletes, successful athletes would show lower late-race velocity loss and more stable or advantageous in-race positions, particularly in the later stages of the race; (2) differences in velocity and place between successful and non-successful athletes would emerge earlier in heats, whereas in finals these differences would be more concentrated in the closing stages; and (3) compared with men, women would exhibit greater overall stability in both velocity and place distribution. For the exact magnitude and timing of differences between successful and non-successful groups across specific scenarios, the analyses were exploratory.

## 2 Materials and methods

### 2.1 Ethical consideration

Data for this study were obtained from publicly available resources. Therefore, no ethical approval or informed consent was required. This study complies with the ethical standards recognized by the Declaration of Helsinki.

### 2.2 Participants

Race results were collected from the men's and women's 3000 m steeplechase events at 3 World Championships (2022, 2023, 2025) and 1 Olympic Games (Tokyo, 2020). The raw data were obtained from World Athletics official result sheets and split-time files. After excluding athletes who did not finish or were disqualified, the initial sample comprised 187 men's and 202 women's performances.

Following the approach of Filipas,^10^ outlier screening of finishing times was conducted using boxplots. Performances whose times deviated from the median by more than 1.5 interquartile ranges (IQR) were considered extremely slow and were removed, resulting in the exclusion of 3 men and 6 women. The same procedure was applied to the relative 100-m velocity variables (*V*_*rel100*_–*V*_*rel3000*_). Observations whose values deviated from the median by more than 3 IQR and were confirmed by manual checking to reflect timing errors were excluded, yielding an additional 10 men and 31 women. In total, race data from 174 men performances and 165 women performances in different competitions were retained for subsequent analyses.

### 2.3 Grouping

Athletes were classified as successful or non-successful according to their finishing place. In the finals, competitors were divided into a Top-8 group (30 men, 27 women) and a Non-Top-8 group (25 men, 23 women). In the heats, athletes were divided into a Qualified group (50 men, 50 women) and a Non-qualified group (69 men, 65 women) based on whether they advanced to the final.

1 male and 1 female athlete received a “qR” mark in the heats, indicating that they were advanced to the final by the referee after being impeded by other competitors. These athletes officially completed the heats and were therefore retained in the dataset in accordance with the official race results. However, because their advancement did not result from the standard time- or place-based qualification process, they were not classified as performance-based qualifiers in the heat analysis. They were therefore classified in the Non-qualified group for the purpose of comparing standard qualification outcomes.

Because some athletes appeared in more than one race round or competition, the distribution of performances, unique athletes, and repeated entries was summarized before inferential modeling (Table 1).

**Table 1:** Distribution of performances, unique athletes, and repeated entries.

Sex	Round	Group	Performances	Unique athletes	Athletes with repeated entries	Repeat-entry performances	Maximum entries per athlete
Men	Final	Top 8	30	20, 66.7%	6, 30.0%	16, 53.3%	4
		Non-Top 8	25	23, 92.0%	2, 8.7%	4, 16.0%	2
	Heats	Qualified	50	37, 74.0%	7, 18.9%	20, 40.0%	4
		Non-qualified	69	49, 71.0%	16, 32.7%	36, 52.2%	4
Women	Final	Top 8	27	20, 74.1%	6, 30.0%	13, 48.1%	3
		Non-Top 8	23	21, 91.3%	2, 9.5%	4, 17.4%	2
	Heats	Qualified	50	35, 70.0%	12, 34.3%	27, 54.0%	3
		Non-qualified	65	49, 75.4%	12, 24.5%	28, 43.1%	3

### 2.4 Analytical approach and pacing indicators

This observational study described velocity and place distribution patterns during the 3000 m steeplechase and compared these patterns between successful and non-successful athletes. Split velocities were first calculated from segment times, and a series of derived indices were then used to characterize velocity stability. To compare male and female pacing profiles, velocities were expressed as a percentage of each athlete's mean race velocity. Because the number of starters differed across races, changes in place were examined using relative place, defined as

$$\text{Relative}\;\text{place}=\frac{\text{rank}-1}{N-1}$$

where rank is the athlete's place at a given split and N is the number of finishers in that race. To quantify the magnitude of velocity changes, two indices were used: Relative Peak Velocity

$$V_{\text{peak}.\text{rel}}=V_{\text{peak}}/V_{\text{mean}}$$

and the Relative Amplitude of Velocity Variation

$$\text{RAVV}=\frac{V_{\text{peak}}-V_{\min}}{V_{\text{mean}}}\times 100\%$$

To characterize late-race changes in velocity over the final 600 m, we employed the Late-race Deceleration Index

$$\text{LDI}=\frac{V_{\text{peak}}-V_{\text{last}600}}{V_{\text{peak}}}\times 100\%$$

and C_last600_ defined as the slope of the velocity–distance curve over the last 600 m.

Pacing stability was further described using three complementary indices: the Coefficient of Variation (CV),^11^ the Quotient of Deviations of Velocity (QVD),^12^ and the Root Mean Square of Successive Differences (RMSSD).^13^ Although all three capture deviations of segmental velocities from the mean race velocity, they were analyzed together because CV reflects overall variability driven by large fluctuations, QVD provides an easily interpretable estimate of the average percentage deviation across all segments, and RMSSD is more sensitive to short-term, segment-to-segment fluctuations in velocity. In addition, we proposed a novel index, Velocity Crossing Count (VCC), defined as the number of intersections between an athlete's velocity curve and their mean race-velocity line, to complement the above indices in describing pacing stability.

Because tactics involve not only how velocity is distributed but also how race place is managed, we also examined variability in relative place. By analogy with CV and QVD for velocity, two place-based indices—CV_place_ and QPD (Quotient of Place Deviations)—were computed to quantify the magnitude and pattern of place changes throughout the race.

### 2.5 Statistical analysis

Most variables were reported as mean ± standard deviation (SD), while relative place was summarized as median and IQR. All statistical analyses were conducted using IBM SPSS Statistics, version 31.0 (IBM Corp., Chicago, IL, USA).

The statistical analysis was organized into two complementary parts. First, descriptive race-profile analyses were used to visualize and characterize the observed velocity and place distributions across all retained performances. Pacing curves were analyzed using a mixed-design ANOVA on relative 100-m split velocities, with distance (30 × 100-m splits) as the within-subject factor and sex as the between-subject factor. Mauchly's test of sphericity was applied to the distance factor, and when the assumption was violated, Greenhouse–Geisser corrections were used. When significant between-group differences in cumulative split time were detected at a given distance, that split was considered the point at which the groups became separated during the race. Statistical significance was set at *p* < 0.05. Effect sizes for pairwise comparisons were calculated as Cohen's d and interpreted as trivial (<0.20), small (0.21–0.60), moderate (0.61–1.20), large (1.21–2.00), or very large (≥2.01).^14^ In the descriptive race-profile analyses, only differences that were both statistically significant (*p* < 0.05) and at least moderate in magnitude (|d| > 0.60) were highlighted in the figures and interpreted as visually meaningful race-profile features.

Second, mixed-effects models were used for inferential analyses of summary pacing and place-related indicators while accounting for repeated athlete entries. Because some athletes contributed more than one performance across competitions, athlete identity was included as a random intercept in the mixed-effects models. For indicators related to continuous time, velocity, and place, linear mixed models (LMMs) were fitted separately for each sex-by-round scenario. Success group and competition were specified as fixed effects, and athlete identity was included as a random intercept. Adjusted mean differences between successful and non-successful athletes were reported with 95% confidence intervals (95% CI), *F* values, and *p* values. Adjusted mean differences were expressed as successful minus unsuccessful. For binary success outcomes, generalized linear mixed models (GLMMs) with a binomial distribution and logit link were fitted separately for men's finals, men's heats, women's finals, and women's heats. Athlete identity was specified as a random intercept to account for repeated entries. Because the number of successful outcomes within each sex-by-round scenario was limited, and several pacing and positioning indicators were conceptually overlapping, no more than two primary predictors were entered into each model. LDI was retained in all models because late-race velocity maintenance was a central hypothesis and showed consistent mixed-model differences between successful and unsuccessful athletes. The second predictor was selected according to the dominant tactical feature of each sex-by-round scenario while avoiding redundancy among related variables. Candidate predictor domains, variables considered, variables entered into the final models, and the rationale for inclusion or exclusion are provided in Supplementary Table S2. Vpeak in the women's final model was entered per 0.1 m/s to improve interpretability of the odds ratio. Statistical significance was set at *p* < 0.05.

## 3 Results

The retained dataset included 339 performances from 144 athletes. The mean finishing time in men was 8:27.37 ± 9.43, which was significantly faster than in women (9:23.84 ± 14.69), corresponding to an approximate difference of 10.0% (*p* < 0.001, d = 4.60). Among the men's performances, 14 were season bests (SB) and 6 were personal bests (PB), with finishing times corresponding to 103.21 ± 2.14% of each athlete's PB. In women, 50 performances were SBs and 36 were PBs, with finishing times corresponding to 101.43 ± 4.75% of PB.

**Fig. 1:**
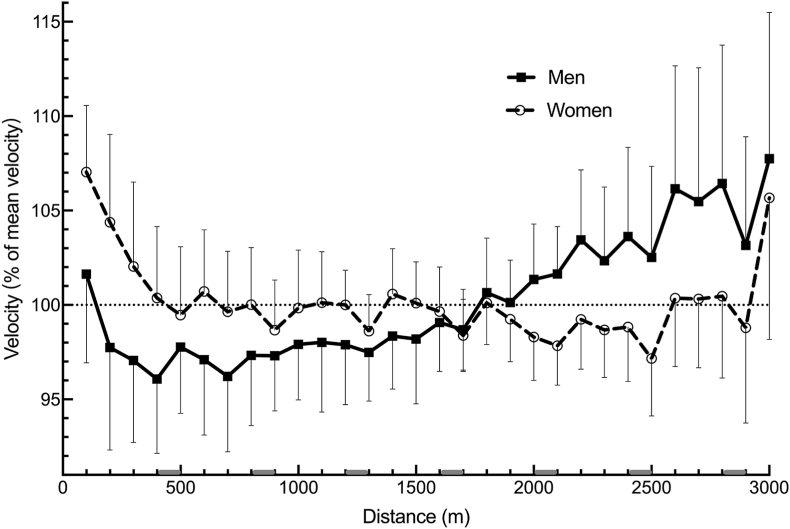
Mean (±SD) 100-m split velocities expressed as a percentage of mean race velocity for men and women in the 3000 m steeplechase. SD error bars are plotted unilaterally: at each split, the group with the higher relative velocity has its SD bar drawn upward, and the group with the lower relative velocity has its SD bar drawn downward. Gray boxes on the x-axis indicate 100-m segments that include the water jump (same convention in subsequent figures). The horizontal dotted line represents 100% of mean race velocity.

A repeated-measures ANOVA on relative 100-m velocities revealed significant main effects of distance and sex, as well as a distance × sex interaction (distance: *F*_(3.88, 1308.77)_ = 94.26, *p* < 0.001, $\eta_{p}^{2}$ = 0.22; sex: *F*_(1, 337)_ = 63.30, *p* < 0.001, $\eta_{p}^{2}$ = 0.16; distance × sex: *F*_(3.88, 1308.77)_ = 74.94, p < 0.001, $\eta_{p}^{2}$ = 0.18). These findings indicate marked within-race fluctuations in velocity and systematic sex-related differences in how pace changed with distance. As illustrated in Fig. 1, both men and women exhibited an overall “fast–slow–fast” (U-shaped) pacing profile across 100-m segments, with women maintaining relatively higher velocity in the early and middle phases of the race, whereas men showed a more pronounced end-spurt over the final part of the race.

**Fig. 2:**
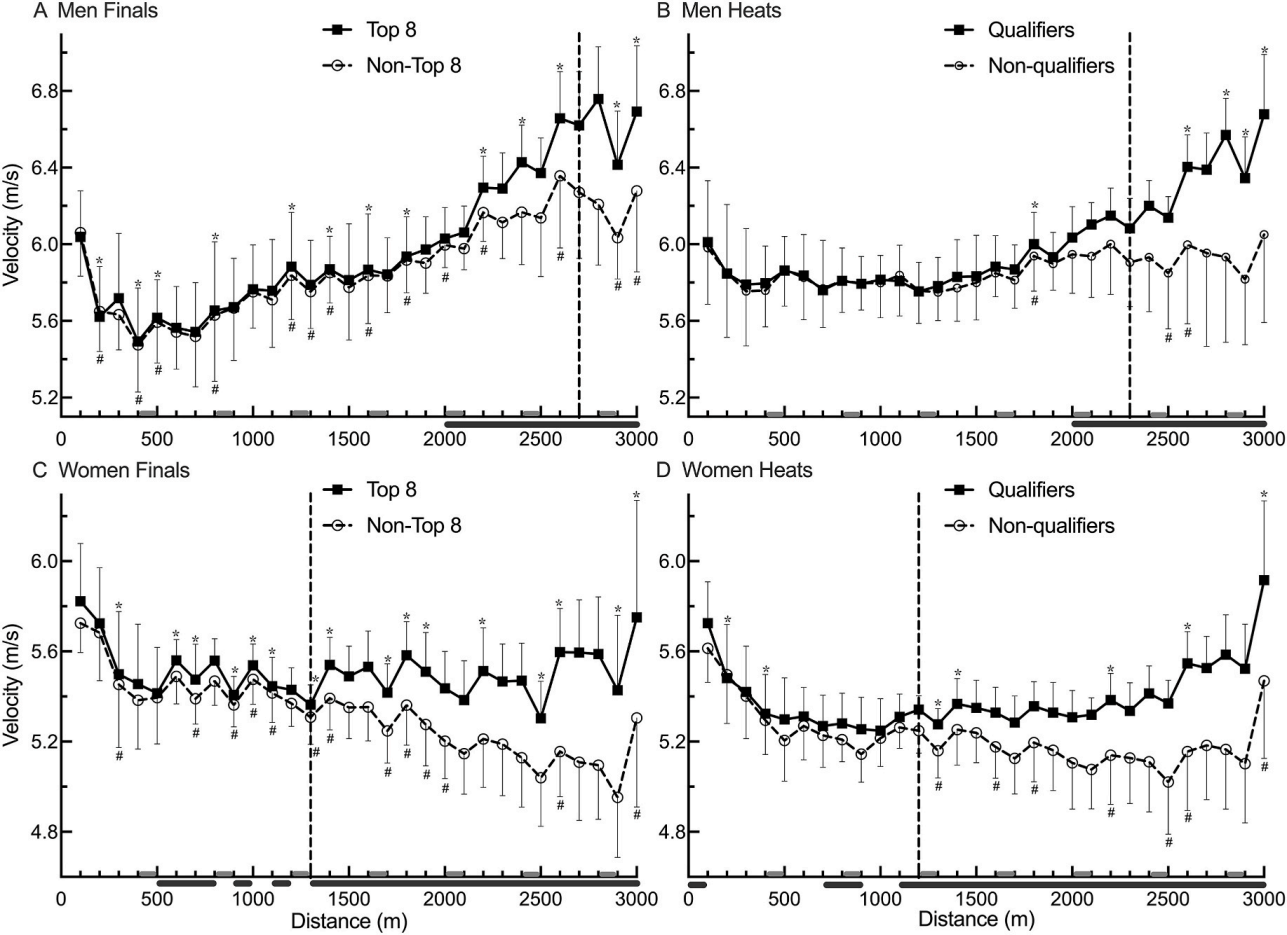
100-m split velocities (mean ± SD) as a function of distance for successful and non-successful athletes in men's and women's heats and finals. SD error bars are plotted unilaterally, with the successful group shown upward and the non-successful group downward. * and hash # indicate within-group changes in velocity compared with the preceding 100-m segment for the successful and non-successful groups respectively. Vertical dashed lines mark the first split at which cumulative race time differed between groups. Gray bands under the x-axis indicate distance ranges where split velocities differed between groups.

Fig. 2 illustrates the 100-m velocity curves for successful and non-successful athletes in men's and women's heats and finals, with annotations indicating splits where: (a) between-group differences in velocity were observed, (b) cumulative race time first differed between groups, and (c) within-group changes between adjacent splits were observed. Overall, the pacing profiles followed a “fast–slow–fast” pattern, with an initial deceleration after the start, a relatively steady mid-race phase, and a marked increase in velocity toward the end. Men showed an earlier and more pronounced late-race acceleration, whereas women maintained a longer period of mid-race steadiness but exhibited earlier between-group differences in velocity. In both sexes, the point at which groups began to diverge in heats occurred earlier than in finals.

In the men's events, between-group differences in split velocity were concentrated in the latter part of the race. In both heats and finals, differences between successful and non-successful athletes emerged between 2000 m and 3000 m, corresponding to the segment from the fifth water jump to the finish, when late-race acceleration occurs. The first split at which cumulative time differed between groups was 2700 m in the final and 2300 m in the heats, located on the straights before the fifth and sixth water jumps, respectively. Regarding within-group velocity changes between adjacent splits, the number of highlighted change points was similar between groups in the men's final (13 for the Top 8 group vs 14 for the Non-Top 8 group), and these were distributed across the entire race. In the heats, the total number of change points was lower than in the final, with more changes in the Qualified group (5) than in the Non-qualified group (3), mainly clustered in the closing stages.

In the women's events, between-group differences in split velocity appeared earlier. In the women's final, between-group differences emerged immediately after the first water jump, disappeared around the second and third water jumps, and then reappeared and persisted until the finish. In the heats, between-group differences appeared on the straight before the second water jump, disappeared briefly after clearing the water jump, and then re-emerged on the straight before the third water jump, remaining evident through to the finish. The first between-group difference in cumulative time occurred at 1300 m in the final and 1200 m in the heats, around the third water jump. For within-group changes between adjacent splits, the Top 8 group in the women's final showed more change points than the Non-Top 8 group (16 vs 13). In the heats, both the Qualified and Non-qualified groups showed the same total number of highlighted change points (7), but these were distributed differently: changes in the Qualified group were spread across the entire race, whereas those in the Non-qualified group were concentrated in the mid-to-late stages and were of greater magnitude.

**Fig. 3:**
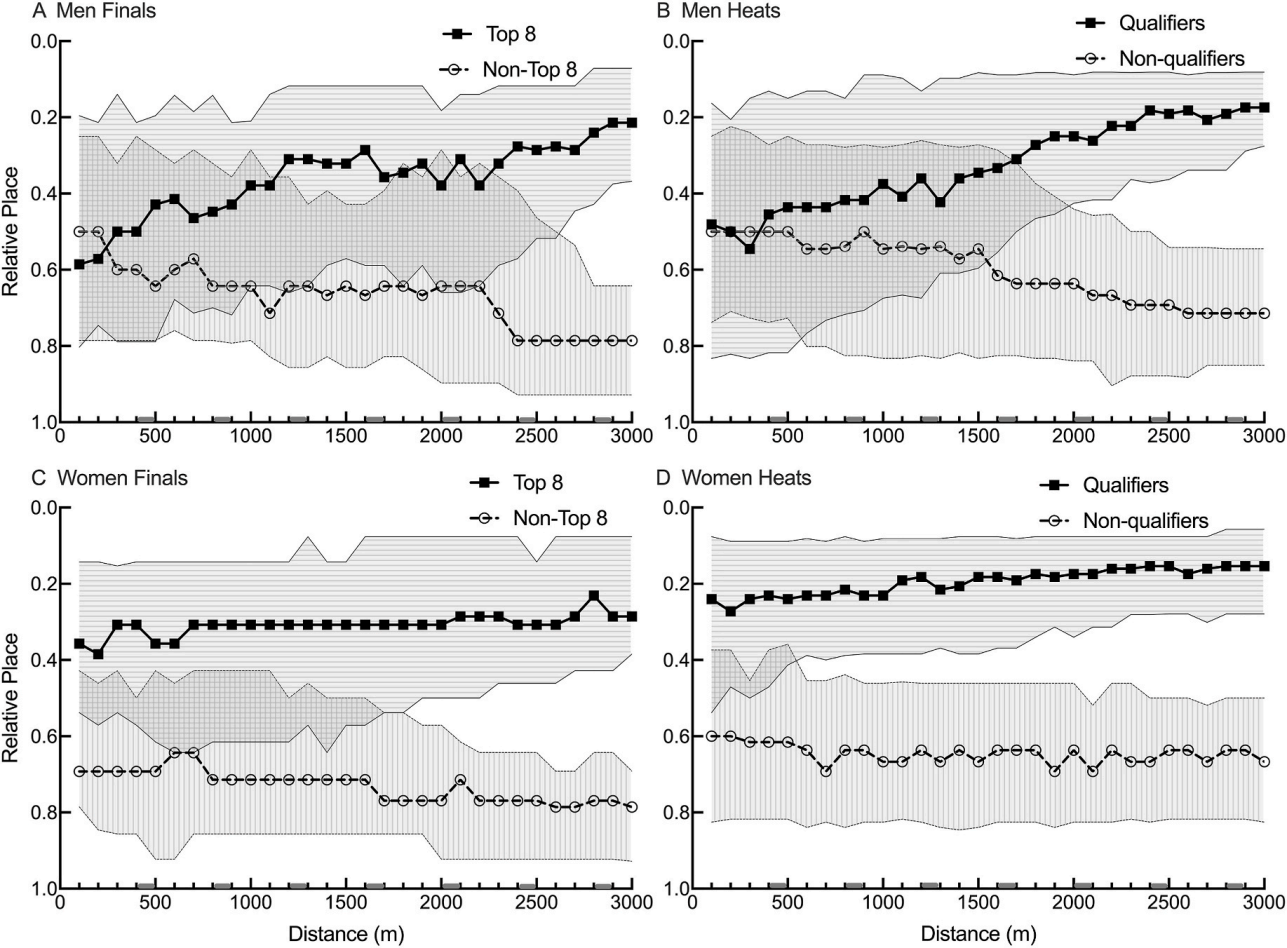
Median relative place as a function of distance in men's and women's 3000 m steeplechase heats and finals. Shaded bands with horizontal hatching indicate the interquartile range for the successful group, and shaded bands with vertical hatching indicate the interquartile range for the non-successful group.

Fig. 3 illustrates the evolution of median relative place with interquartile ranges across distance for successful and non-successful athletes in the four race scenarios. In the men's races, the two groups showed overlapping places in the early phase, then gradually separated after the first water jump, followed by only modest fluctuations in rank. A stable separation between groups was established from approximately 2600 m in the final and 2000 m in the heats. In the women's races, the median race places of the two groups became stratified shortly after the start, showing an early settling of race order, with stable separation emerging from around 1800 m in the final and 600 m in the heats. Overall, changes in relative place were smaller among women than among men.

Table 2 summarizes the LMM-adjusted differences between successful and non-successful athletes for indicators related to time, velocity, and place across the four scenarios. More details are provided in Supplementary Table S1. After accounting for repeated athlete entries and competition effects, successful athletes had higher V_mean_, V_peak_, and lower LDI than non-successful athletes in all four scenarios. These results indicate that successful performances were consistently characterized by superior overall velocity, greater peak velocity, and less late-race velocity loss.

**Table 2:** Summary of LMM-adjusted differences between successful and non-successful athletes.

Variable	Men × Final	Men × Heat	Women × Final	Women × Heat
Finish time	↓***	↓***	↓***	↓***
PB%	–	↓**	–	–
SB%	–	↓***	↓***	–
V_mean_	↑***	↑***	↑***	↑***
V_min_	–	↑***	↑***	↑***
V_peak_	↑***	↑***	↑***	↑***
V_peak.rel_	↑***	↑**	–	↑*
RAVV	↑***	–	–	–
LDI	↓**	↓***	↓**	↓***
C_last600_	↑*	↑***	–	–
CV	↑***	↑***	↓*	–
QVD	↑***	↑***	↓**	–
RMSSD	–	–	↑*	–
VCC	↓**	↓**	↑**	–
CV_place_	–	↑***	↑**	↑***
QPD	–	↑***	↑**	↑***

Arrows indicate the direction of the successful group relative to the non-successful group based on LMM-adjusted comparisons. ↑ indicates higher values in successful athletes; ↓ indicates lower values in successful athletes; – indicates no statistically significant difference. *p < 0.05, **p < 0.01, ***p < 0.001. Full descriptive values, adjusted mean differences, 95% confidence intervals, *F* values, and exact *p* values are provided in Supplementary Table S1.

In men, successful athletes generally showed greater velocity modulation, as reflected by higher V_peak.rel_, C_last600_, CV, QVD together with lower VCC. In addition, successful athletes showed higher RAVV in the finals, and higher V_min_, CV_place_ and QPD in the heats. In women, successful athletes also showed higher V_min_, CV_place_, and QPD. Furthermore, successful women had higher V_peak.rel_ in the heats, and higher RMSSD and VCC as well as lower CV and QVD in the finals.

Table 3 shows the GLMM results for successful outcomes after accounting for repeated athlete entries. In men's finals, lower LDI was associated with a higher likelihood of success, whereas CV showed a positive but not significant association. In men's heats, both lower LDI and higher CV were associated with successful qualification. In women's finals, lower LDI and higher VPEAK, expressed per 0.1 m/s, were associated with success. In women's heats, lower LDI and higher CV_place_ were associated with successful qualification. Overall, LDI was the most consistent correlate of success across all four sex-by-round scenarios.

**Table 3:** GLMM results for successful vs non-successful outcomes across the four race scenarios.

Scenario	Predictor	β	SE	OR (95% CI)	*p*
Men × Final	LDI	−0.441	0.204	0.644 (0.427, 0.969)	**0.035**
	CV	0.317	0.169	1.373 (0.978, 1.929)	0.067
Men × Heat	LDI	−0.355	0.090	0.701 (0.587, 0.838)	**<0.001**
	CV	0.647	0.198	1.909 (1.290, 2.826)	**0.001**
Women × Final	LDI	−0.387	0.136	0.679 (0.517, 0.892)	**0.006**
	VPEAK	1.348	0.407	3.851 (1.697, 8.740)	**0.002**
Women × Heat	LDI	−0.156	0.075	0.856 (0.738, 0.992)	**0.039**
	CV_place_	0.066	0.018	1.068 (1.031, 1.106)	**<0.001**

ORs are expressed for successful outcomes. VPEAK represents V_peak_ per 0.1 m/s.

## 4 Discussion

This study showed that in Olympic Games and World Championships, both men's and women's 3000 m steeplechase races exhibited an overall fast–slow–fast, inverted parabolic pacing profile. This pattern is broadly consistent with previous findings in the marathon, 5000 m, and 10,000 m events.^11^ Male athletes typically started at a moderately fast velocity, showed a brief deceleration around the first water jump, then gradually increased velocity in the middle and late stages and reached their highest velocity just before the finish. Female athletes ran relatively faster at the start and during the final sprint after the last water jump, but adopted a more even pace through the middle of the race, resulting in a profile closer to even pacing. This sex difference aligns with the frequently reported tendency for women to pace more evenly in endurance events,^15^ but it differs from the pacing profiles reported by Hanley^2^ for the Olympic 3000 m steeplechase in Beijing and Rio. One possible explanation is that the two Olympics analyzed by Hanley were held shortly after the women's 3000 m steeplechase was introduced into the Olympic program, when optimal pacing tactics were still evolving. In contrast, the four global championships covered in the present study span more than a decade from then, during which time elite female steeplechasers may have converged toward more mature and stable pacing tactics. In addition, women in our sample achieved personal-best and season-best performances more frequently than men, which indirectly supports the notion that, when the objective is to minimize finishing time, relatively even pacing may be advantageous.^16^

These sex-specific differences in pacing were also evident when comparing successful and non-successful athletes. As shown in Fig. 2, in the men's races, the pacing curves of successful and non-successful athletes largely overlapped during the early and middle stages, and the most pronounced differences emerged in the final third of the race. The decisive factor appeared to be that successful athletes were able to produce and sustain higher velocities in the last few laps, particularly after the fifth water jump, whereas non-successful athletes showed limited capacity to accelerate in the same phase. In the women's races, differences between successful and non-successful athletes emerged earlier, mainly in the early to middle segments: non-successful athletes showed an earlier decline in velocity and were unable to maintain the pace established after the start. Overall, successful male athletes tended to rely more on high-intensity late-race acceleration, whereas successful female athletes depended more on sustaining a relatively high and stable velocity over a longer distance. This suggests that the pathway to success differs between men and women in the steeplechase.

The relationship between pacing and success also showed round-dependent characteristics. In the men's events, the overall pacing pattern was similar across performance levels and rounds: athletes tended to increase velocity progressively after the first water jump, with the most visible decelerations occurring around the final two water jumps. However, the successful and non-successful groups differed in how strongly they could re-accelerate in the latter part of the race. In the heats, non-successful athletes showed less late-race acceleration, leading to an earlier divergence in cumulative time between groups—approximately 400 m earlier than in the final. This suggests that, in men's races, differences in late-race acceleration capacity became evident earlier under qualification conditions. In the women's events, pacing was characterized by a recurring pattern of acceleration on the straight after each water jump followed by progressive deceleration over the next three 100-m segments. The divergence points in cumulative time between successful and non-successful athletes occurred at approximately 1300 m in the final and 1200 m in the heats, which are relatively close. However, the between-group differences in velocity during the latter stages were clearly larger in the final than in the heats, with non-top 8 in the final exhibiting more pronounced late-race deceleration. Sex-specific fatigability is complex and task-dependent, and may be influenced by exercise intensity, muscle group involvement, training history, and recovery status.^17^ Therefore, the greater late-race deceleration observed among women in the final should not be attributed to sex alone. One possible explanation is that some athletes may not have fully recovered from the demands of the heats, but this remains speculative and requires confirmation in future studies incorporating physiological and recovery-related measurements.

The patterns of separation in relative place were broadly consistent with those observed in cumulative time. As shown in Fig. 3, in the men's races, there were frequent rank crossovers between groups during the start and early to middle phases, and successful athletes were not in absolute leading places from the outset. They gradually moved forward after the first water jump and established a stable separation zone in the middle-to-late stages. In the women's races, the place hierarchy emerged earlier: the median relative place of the successful group stabilized near the front soon after the start, and the stable separation between groups was already evident in the early–middle segments. Together with the CV_place_ and QPD results, which showed more frequent place adjustments among successful athletes in all scenarios except the men's final, these findings indicate that successful steeplechasers do not simply run in a fixed place. Instead, they maintain an overall advantageous place while making proactive adjustments in key segments to secure and reinforce favorable race places. This is consistent with previous work showing that high-level athletes tend to implement controlled changes in pace and place within the lead pack rather than relying solely on a late all-out sprint.^18^ Such a tactic may help them reduce unnecessary physical contact, preserve room to maneuver around barriers and competitors, and gradually restrict their opponents' tactical options and expectations of success.

The present study characterized steeplechase tactics across four dimensions: velocity, late-race performance, velocity stability, and place variability. After accounting for repeated athlete entries in the mixed-model analyses, the most robust findings were that successful athletes consistently achieved higher V_mean_ and V_peak_ and lower LDI across all scenarios. LDI and Cl_ast600_ together reflect the ability to maintain or re-express velocity in the final 600 m, but LDI showed the most consistent association with success. This supports late-race velocity maintenance as the most consistent tactical feature distinguishing successful performances, in line with findings from other running events.^9^ With respect to velocity stability, CV, QVD, RMSSD, and VCC capture distinct aspects of pacing variability. Successful male athletes generally showed higher CV and QVD, supporting the interpretation that male success is associated with larger global velocity modulation. In contrast, successful women in the final showed lower CV and QVD but higher RMSSD and VCC, suggesting that women's final success may depend more on maintaining a high overall velocity with controlled local adjustments rather than large global fluctuations. These findings reinforce that effective pacing is not equivalent to minimizing all velocity changes. Instead, the optimal balance between stability and variability appears to differ by sex and race round.

GLMMs were used to identify the key tactical features associated with success after accounting for repeated athlete entries. Across all four models, LDI remained negatively associated with success, underscoring that limiting late-race velocity loss or retaining the capacity to accelerate in the closing stages is a common hallmark of successful 3000 m steeplechase performance under championship conditions. Beyond LDI, the second predictor showed scenario specificity. In men's heats, CV was positively associated with qualification, whereas in men's finals the positive association between CV and success did not reach statistical significance after accounting for repeated athlete entries. In women's finals, VPEAK was positively associated with success, indicating the importance of peak velocity capacity in highly competitive finals. In women's heats, CV_place_ was positively associated with qualification, implying that active positional adjustment within the pack may be conducive to reaching the final. These models should be interpreted as explanatory association models rather than validated prediction tools for individual outcomes.

The main strengths of this study lie in the use of a large, contemporary, and high-resolution dataset, and in the integration of velocity distribution, place distribution, and multiple pacing-stability indices within a single analytical framework. Building on traditional indicators, we additionally introduced novel metrics such as VCC, CV_place_, and QPD, thereby examining steeplechase tactics from a combined “velocity–place–stability” perspective and characterizing the tactical behaviors of successful and non-successful athletes in world-class competitions. However, several limitations should be acknowledged. All participants were Olympic and World Championship steeplechasers, so the findings may not generalize to youth athletes or those at lower competitive levels, and the observed tactics may not be directly applicable to other race formats. In addition, although 100-m split data provide the highest publicly available resolution for these competitions, the segments are not mechanically identical because the steeplechase lap structure differs from a standard 400-m flat lap, so the number and relative position of barriers and the water jump vary across the lap. Therefore, the present analysis cannot isolate the independent effect of each barrier or water jump on velocity change. Moreover, the analyses were based solely on official timing and split-place records and did not include physiological measures such as heart rate, blood lactate, or power output, which prevents direct inferences about the metabolic costs of different pacing tactics. Future research could seek to replicate this analytical framework across different performance levels, age groups, and competition types, and to combine split data with wearable-derived locomotor and physiological measures to explore the physiological responses and fatigue trajectories associated with alternative pacing tactics in the steeplechase.

## 5 Practical applications

For male 3000 m steeplechasers, in addition to traditional aerobic endurance and hurdling technique training, particular emphasis should be placed on high-intensity variable-pace and sprint sessions that simulate the closing stages of major championships. The training should target the ability to re-accelerate rapidly after the water jump and to execute repeated accelerations within the final 1000 m, while tactical drills should help athletes control their pace in the early and middle phases of the race to avoid excessive early expenditure and preserve sufficient reserves for the final sprint. For female athletes, greater emphasis should be placed on pacing stability and rhythm control in the early and middle parts of the race, aiming to secure an appropriate place shortly after the start and then maintain a moderately high, sustainable velocity within a favorable pack.

## CRediT authorship contribution statement

**Fangzhou He:** Conceptualization, Data curation, Formal analysis, Investigation, Methodology, Software, Visualization, Writing – original draft; **Anning Guo:** Data curation, Investigation; **Mujuan Zhao:** Data curation, Formal analysis; **Jiqing Cui:** Data curation, Validation; **Włodzimierz S. Erdmann:** Conceptualization, Methodology, Project administration, Supervision, Writing – review & editing.

## Funding

No financial or material support of any kind was received for the work described in this article.

## Declaration of competing interest

The authors declare that they have no conflict of interest relating to the material presented in this article.

## References

[1] IAAF. *Book of Rules* (2025). Official documents. https://worldathletics.org/about-iaaf/documents/book-of-rules.

[2] Hanley B., Williams E.L. (2020). Successful Pacing Profiles of Olympic Men and Women 3,000 m Steeplechasers. Front Sports Act Living.

[3] Cook C., Chen G., Hager W.W., Lenhart S. (2023). Optimally controlling nutrition and propulsion force in a long distance running race. Front Nutr.

[4] Noorbergen O.S., Konings M.J., Micklewright D., Elferink-Gemser M.T., Hettinga F.J. (2016). Pacing behavior and tactical positioning in 500- and 1000-m short-track speed skating. Int J Sports Physiol Perform.

[5] Hanley B., Stellingwerff T., Hettinga F.J. (2019). Successful pacing profiles of olympic and IAAF world championship middle-distance runners across qualifying rounds and finals. Int J Sports Physiol Perform.

[6] Rodriguez L., Veiga S. (2018). Effect of the pacing strategies on the open-water 10-km world swimming championships performances. Int J Sports Physiol Perform.

[7] Casado A., Ranieri L.E., Hanley B., Foster C., González-Mohíno F. (2024). Differences in pacing behaviour between global championship medal performances and world records in men's and women's middle- and long-distance running track events. J Sports Sci.

[8] Konings M.J., Hettinga F.J. (2018). The impact of different competitive environments on pacing and performance. Int J Sports Physiol Perform.

[9] Casado A., Hanley B., Jiménez-Reyes P., Renfree A. (2021). Pacing profiles and tactical behaviors of elite runners. J Sport Health Sci.

[10] Filipas L., La Torre A., Hanley B. (2021). Pacing profiles of olympic and IAAF world championship long-distance runners. J Strength Condit Res.

[11] Hettinga F.J., Edwards A.M., Hanley B. (2019). The science behind competition and winning in athletics: using world-level competition data to explore pacing and tactics. Front Sports Act Living.

[12] Erdmann W.S., Lipinska P. (2013). Kinematics of marathon running tactics. Hum Mov Sci.

[13] Boullosa D., Patrocínio E., Renfree A., Casado A., Hanley B., Foster C. (2023). Short-term speed variability as an index of pacing stochasticity in athletic running events. J Funct Morphol Kinesiol.

[14] Hopkins W.G., Marshall S.W., Batterham A.M., Hanin J. (2009). Progressive statistics for studies in sports medicine and exercise science. Med Sci Sports Exerc.

[15] Deaner R.O., Carter R.E., Joyner M.J., Hunter S.K. (2015). Men are more likely than women to slow in the marathon. Med Sci Sports Exerc.

[16] Abbiss C.R., Laursen P.B. (2008). Describing and understanding pacing strategies during athletic competition. Sports Med.

[17] Tornero-Aguilera J.F., Jimenez-Morcillo J., Rubio-Zarapuz A., Clemente-Suárez V.J. (2022). Central and peripheral fatigue in physical exercise explained: a narrative review. Int J Environ Res Publ Health.

[18] Smits B.L.M., Pepping G.J., Hettinga F.J. (2014). Pacing and decision making in sport and exercise: the roles of perception and action in the regulation of exercise intensity. Sports Med.

