# Supplementary material for: Pace and place tactics of top performers in the world's 3000 m steeplechase competitions
Source: J Exerc Sci Fit. 2026 Jul 28;24(4):200497. doi: 10.1016/j.jesf.2026.200497 (PMC13452380; doi:10.1016/j.jesf.2026.200497)
Supplement: Supplementary file 1 [file mmc1.docx]

Supplementary Table S1. Time-, velocity-, and place-related indices (mean ± SD) for men and women in the successful and non-successful groups

| Variable | Scenario | Successful (mean ± SD) | Unsuccessful (mean ± SD) | Adjusted mean difference (95% CI) | *F* | *p* |
| --- | --- | --- | --- | --- | --- | --- |
| Finish time | **Men×Final** | **8:22.57±10.48** | **8:31.21±7.63** | **-7.67 (-9.83, -5.51)** | **50.904** | **<0.001** |
|  | **Men×Heat** | **8:21.08±6.15** | **8:32.63±7.67** | **-11.66 (-13.93, -9.38)** | **103.224** | **<0.001** |
|  | **Women×Final** | **9:05.42±7.96** | **9:25.83±10.42** | **-20.39 (-25.05, -15.73)** | **78.404** | **<0.001** |
|  | **Women×Heat** | **9:17.11±4.91** | **9:35.97±11.92** | **-18.64 (-22.10, -15.19)** | **114.428** | **<0.001** |
| PB% | Men×Final | 103.77±2.47 | 103.71±2.24 | -0.13 (-0.69, 0.43) | 0.216 | 0.644 |
|  | **Men×Heat** | **102.51±1.90** | **103.31±2.02** | **-0.91 (-1.49, -0.34)** | **9.992** | **0.002** |
|  | Women×Final | 98.60±10.46 | 102.41±2.19 | -3.94 (-8.51, 0.64) | 3.004 | 0.090 |
|  | Women×Heat | 101.91±1.99 | 101.88±2.15 | -0.27 (-1.06, 0.51) | 0.474 | 0.492 |
| SB% | Men×Final | 103.06±2.38 | 103.51±2.10 | -0.51 (-1.10, 0.08) | 3.016 | 0.089 |
|  | **Men×Heat** | **101.87±2.23** | **102.90±2.01** | **-1.13 (-1.77, -0.48)** | **12.029** | **<0.001** |
|  | **Women×Final** | **99.86±1.19** | **102.06±1.64** | **-2.32 (-3.11, -1.53)** | **35.361** | **<0.001** |
|  | Women×Heat | 101.08±1.87 | 101.02±2.15 | 0.08 (-0.67, 0.83) | 0.044 | 0.834 |
| V_mean_ | **Men×Final** | **5.97±0.13** | **5.87±0.09** | **0.09 (0.07, 0.12)** | **49.175** | **<0.001** |
|  | **Men×Heat** | **5.99±0.07** | **5.85±0.09** | **0.14 (0.11, 0.16)** | **106.271** | **<0.001** |
|  | **Women×Final** | **5.50±0.08** | **5.30±0.10** | **0.20 (0.15, 0.24)** | **81.238** | **<0.001** |
|  | **Women×Heat** | **5.39±0.05** | **5.21±0.11** | **0.17 (0.14, 0.20)** | **120.711** | **<0.001** |
| V_min_ | Men×Final | 5.38±0.25 | 5.40±0.21 | -0.02 (-0.09, 0.06) | 0.251 | 0.619 |
|  | **Men×Heat** | **5.56±0.22** | **5.39±0.24** | **0.17 (0.10, 0.25)** | **19.233** | **<0.001** |
|  | **Women×Final** | **5.08±0.23** | **4.85±0.22** | **0.25 (0.12, 0.37)** | **15.924** | **<0.001** |
|  | **Women×Heat** | **5.09±0.10** | **4.88±0.17** | **0.21 (0.16, 0.26)** | **69.287** | **<0.001** |
| V_peak_ | **Men×Final** | **6.92±0.20** | **6.63±0.25** | **0.30 (0.20, 0.39)** | **43.149** | **<0.001** |
|  | **Men×Heat** | **6.75±0.21** | **6.47±0.30** | **0.28 (0.19, 0.38)** | **32.803** | **<0.001** |
|  | **Women×Final** | **6.08±0.17** | **5.82±0.14** | **0.26 (0.17, 0.40)** | **39.052** | **<0.001** |
|  | **Women×Heat** | **6.04±0.23** | **5.76±0.19** | **0.28 (0.20, 0.35)** | **53.334** | **<0.001** |
| V_peak.rel_ | **Men×Final** | **115.88±4.88** | **112.96±5.40** | **3.23 (1.96, 4.50)** | **25.916** | **<0.001** |
|  | **Men×Heat** | **112.79±3.98** | **110.53±4.54** | **2.20 (0.64, 3.76)** | **7.830** | **0.006** |
|  | Women×Final | 110.47±2.99 | 109.84±2.76 | 0.75 (-0.84, 2.33) | 0.907 | 0.346 |
|  | **Women×Heat** | **112.23±4.24** | **110.56±3.53** | **1.62 (0.20, 3.04)** | **5.089** | **0.026** |
| RAVV | **Men×Final** | **25.76±6.88** | **21.07±7.55** | **5.02 (2.95, 7.09)** | **23.656** | **<0.001** |
|  | Men×Heat | 19.99±5.85 | 18.53±7.14 | 1.34 (-1.01, 3.68) | 1.280 | 0.260 |
|  | Women×Final | 18.06±4.62 | 18.39±4.76 | -0.76 (-3.30, 1.78) | 0.364 | 0.550 |
|  | Women×Heat | 17.65±4.81 | 16.88±4.81 | 0.69 (-1.05, 2.43) | 0.617 | 0.434 |
| LDI | **Men×Final** | **4.75±1.61** | **6.21±2.05** | **-1.42 (-2.44, -0.41)** | **7.947** | **0.007** |
|  | **Men×Heat** | **4.86±2.08** | **8.23±4.49** | **-3.15 (-4.44, -1.86)** | **23.369** | **<0.001** |
|  | **Women×Final** | **8.74±3.41** | **12.24±3.99** | **-3.58 (-5.70, -1.46)** | **11.582** | **0.001** |
|  | **Women×Heat** | **7.64±2.75** | **9.98±3.99** | **-2.36 (-3.67, -1.05)** | **12.763** | **<0.001** |
| C_last600_ (×10^4^) | **Men×Final** | **2.90±7.40** | **-0.94±9.72** | **4.68 (0.07, 9.29)** | **4.163** | **0.047** |
|  | **Men×Heat** | **7.73±5.90** | **1.27±8.35** | **6.19 (3.48, 8.91)** | **20.408** | **<0.001** |
|  | Women×Final | 4.92±10.23 | 2.02±7.00 | 3.74 (-1.19, 8.68) | 2.337 | 0.134 |
|  | Women×Heat | 7.79±6.83 | 5.90±5.40 | 1.68 (-0.63, 3.98) | 2.079 | 0.152 |
| CV | **Men×Final** | **6.99±2.06** | **5.43±2.16** | **1.52 (1.12, 1.92)** | **57.996** | **<0.001** |
|  | **Men×Heat** | **5.12±1.29** | **4.34±1.26** | **0.76 (0.35, 1.17)** | **13.224** | **<0.001** |
|  | **Women×Final** | **3.91±0.72** | **4.43±1.24** | **-0.57 (-1.08, -0.06)** | **5.087** | **0.029** |
|  | Women×Heat | 3.78±0.82 | 3.81±1.02 | -0.09 (-0.45, 0.26) | 0.275 | 0.601 |
| QVD | **Men×Final** | **5.59±1.78** | **4.38±1.83** | **1.13 (0.84, 1.42)** | **61.212** | **<0.001** |
|  | **Men×Heat** | **4.05±1.06** | **3.36±0.99** | **0.67 (0.36, 0.99)** | **17.711** | **<0.001** |
|  | **Women×Final** | **2.89±0.51** | **3.48±1.14** | **-0.63 (-1.06, -0.21)** | **9.093** | **0.004** |
|  | Women×Heat | 2.70±0.56 | 2.90±0.85 | -0.27 (-0.55, 0.02) | 3.532 | 0.064 |
| RMSSD | Men×Final | 0.24±0.07 | 0.21±0.04 | 0.03 (-0.01, 0.06) | 2.687 | 0.107 |
|  | Men×Heat | 0.21±0.06 | 0.22±0.11 | -0.01 (-0.04, 0.03) | 0.205 | 0.651 |
|  | **Women×Final** | **0.20±0.04** | **0.17±0.06** | **0.03 ( 0.01, 0.06)** | **5.016** | **0.030** |
|  | Women×Heat | 0.17±0.05 | 0.17±0.05 | 0.00 (-0.01, 0.02) | 0.170 | 0.681 |
| VCC | **Men×Final** | **4.50±2.13** | **5.92±3.00** | **-1.43 (-2.38, -0.78)** | **9.117** | **0.004** |
|  | **Men×Heat** | **5.70±3.51** | **7.96±4.12** | **-2.10 (-3.36, -0.84)** | **10.872** | **0.001** |
|  | **Women×Final** | **10.37±2.99** | **7.57±4.19** | **2.75 (0.75, 4.75)** | **7.717** | **0.008** |
|  | Women×Heat | 8.66±3.40 | 8.17±3.92 | 0.51 (-0.87, 1.89) | 0.539 | 0.464 |
| CV_place_ | Men×Final | 45.43±23.17 | 34.29±24.85 | 8.26 (-5.45, 21.98) | 1.467 | 0.232 |
|  | **Men×Heat** | **47.89±20.33** | **31.96±18.67** | **13.93 (6.76, 21.11)** | **14.810** | **<0.001** |
|  | **Women×Final** | **32.98±22.85** | **17.64±13.89** | **14.36 (4.35, 24.37)** | **8.397** | **0.006** |
|  | **Women×Heat** | **36.54±19.75** | **19.27±12.42** | **16.99 (10.89, 23.10)** | **30.534** | **<0.001** |
| QPD | Men×Final | 36.07±18.92 | 27.86±20.86 | 4.98 (-6.20, 16.16) | 0.801 | 0.375 |
|  | **Men×Heat** | **38.77±15.71** | **26.38±15.95** | **11.25 (5.40, 17.10)** | **14.529** | **<0.001** |
|  | **Women×Final** | **24.98±15.13** | **14.07±11.48** | **10.19 (3.06, 17.32)** | **8.348** | **0.006** |
|  | **Women×Heat** | **28.05±15.38** | **15.30±10.84** | **12.73 (7.82, 17.64)** | **26.358** | **<0.001** |

Adjusted mean differences are expressed as successful minus unsuccessful. C_last600_ values are reported as ×10⁴.

Supplementary Table S2. Candidate predictor domains and rationale for variable selection in the GLMMs

| Candidate domain | Variables considered | Variables entered in final GLMMs | Rationale for inclusion or exclusion |
| --- | --- | --- | --- |
| Late-race velocity maintenance | LDI, C_last600_ | LDI in all four models | LDI was retained in all models because late-race velocity maintenance was a central hypothesis of the study and LDI showed consistent mixed-model differences between successful and unsuccessful athletes across all sex-by-round scenarios. C_last600_ was considered but not entered because it partly overlapped conceptually with LDI and showed less consistent group differences. |
| Global pacing variability | CV, QVD, RAVV | CV in men’s final and men’s heat models | CV was selected for the men’s models because it provides a direct and interpretable measure of global pacing variability. QVD and RAVV were considered but not entered simultaneously to avoid redundancy among closely related variability indicators. |
| Peak velocity capacity | V_peak_, Vp_eak.rel_ | V_peak_ in the women’s final model | V_peak_ was selected for the women’s final model because peak velocity capacity represented the clearest speed-related characteristic associated with final success. V_peak.rel_ was considered but not selected because absolute peak velocity was more directly interpretable and less dependent on each athlete’s mean race velocity. |
| Short-term velocity fluctuation | RMSSD, VCC | Not entered in final GLMMs | RMSSD and VCC were considered as descriptors of short-term velocity fluctuation, but they were not entered into the final GLMMs because they were secondary indicators and partly overlapped with broader variability measures such as CV and QVD. |
| Place variability and tactical positioning | CV_place_, QPD | CV_place_ in the women’s heat model | CV_place_ was selected for the women’s heat model because qualification races place greater tactical importance on maintaining and adjusting in-race position. QPD was considered but not entered simultaneously with CV_place_ to avoid redundancy between place-variability indicators. |
| Overall performance level | Finish time, V_mean_, V_min_ | Not entered in final GLMMs | Finish time and V_mean_ were not entered because they are directly related to overall race performance; V_min_ was treated as a descriptive performance indicator rather than a primary tactical predictor. |
| Contextual performance indicators | PB%, SB% | Not entered in final GLMMs | PB% and SB% were treated as contextual indicators of athletes’ performance level relative to their personal or seasonal bests rather than primary pacing or positioning predictors. They were therefore not included in the final GLMMs. |

GLMM = generalized linear mixed model; LDI = late-race deceleration index; CV = coefficient of variation; QVD = quotient of velocity deviations; RAVV = relative amplitude of velocity variation; RMSSD = root mean square of successive differences; VCC = velocity crossing count; QPD = quotient of place deviations.
